# Newly designed retentive posts of mandibular reconstruction plate in oral cancer patients based on preliminary FEM study

**DOI:** 10.1186/s12957-016-1043-x

**Published:** 2016-11-21

**Authors:** Ik Jae Kwon, Mi Young Eo, Sung Jae Park, Soung Min Kim, Jong Ho Lee

**Affiliations:** 1 Department of Oral and Maxillofacial Surgery, Dental Research Institute, School of Dentistry, Seoul National University, 101 Daehak-ro, Jongno-gu, Seoul, 110-768 South Korea; 2 Mediwum Inc., Seoul, South Korea

**Keywords:** Reconstruction plate, Mandibular reconstruction, Dental rehabilitation, Retentive post, Flexible denture

## Abstract

**Background:**

The reconstruction of a large mandibular defect poses a challenging issue in oral cancer ablation surgery. One popular option for mandibular continuity reconstruction after tumor resection involves the use of a reconstruction plate (R-plate), which maintains space and contour without bone harvesting. An R-plate, however, cannot provide final functional loading rehabilitation with implants or dentures.

**Methods:**

We suggest a new method of functional mandibular reconstruction using retentive posts and an upper prosthesis. The finite element method (FEM) was used to optimize the design. Surgery was needed to adapt the retentive posts. Prosthodontic procedures were required for the upper prosthesis.

**Results:**

Eight patients were treated with retentive posts and prostheses. All patients underwent wide resections of the mandible, and reconstruction with an R-plate and microvascular soft tissue transfer. We adapted the retentive posts on an R-plate and fabricated the upper prostheses with a flexible denture or a fixed resin prosthesis. Finally, the patients had functional rehabilitation, which restored proper mastication.

**Conclusions:**

The retentive posts of the R-plate and upper prosthesis allow functional dental rehabilitation without the need for a bone graft. Virtual simulation using FEM will help to design and optimize the retentive posts. Two or three regular size posts are suitable for the quadrant jaw. This first preliminary step will allow improved patient-specific retentive post designs in the near future.

## Background

Reconstruction of a large mandibular defect poses a challenging issue in oral and maxillofacial surgery. Following tumor resection, the use of a reconstruction plate (R-plate) to maintain space and contour without requiring bone harvesting has been a popular option to reconstruct the mandible. For large-volume defects involving the bone and soft tissue, the use of a soft tissue free flap (such as the latissimus dorsi (LD) free flap) and R-plate demonstrated a high success rate and low complication rate [1]. Although an R-plate helps the patient to masticate, swallow, and speak, it cannot provide final functional rehabilitation like that of an implant or denture. If patients underwent jaw reconstruction without a bone graft, they could not receive any dental rehabilitation (including with dentures or dental implants) until now [2].

Some surgeons prefer a staged protocol for dental rehabilitation after an en bloc resection [3]. These surgeons performed an R-plate reconstruction with a secondary bone graft from the iliac crest bone. Finally, the implants were placed and prosthodontic reconstruction was performed. However, there was no way to reconstruct the dental functional rehabilitation including chewing, normal swallowing, and acceptable pronunciations for patients who could not tolerate an additional major surgery.

The fibula free flap is a well-known microvascular free flap used in mandibular reconstruction [4]. Although the fibula free flap is advantageous in dental implant installations, larger soft tissue free flaps (such as the LD) are needed for larger soft tissue defects [1]. Furthermore, if dental rehabilitation is not anticipated in the fibula, then plate reconstruction (using adequate soft tissue) remains a suitable technique to repair segmental defects of the lateral mandible [5]. Mandibular reconstruction with a fibula free flap is not clearly beneficial to that using a reconstruction plate after the segmental resection of the mandible [5]. Therefore, if we can offer dental rehabilitation to patients who have already undergone jaw reconstruction with an R-plate, their quality of life will be improved.

**Fig. 1:**
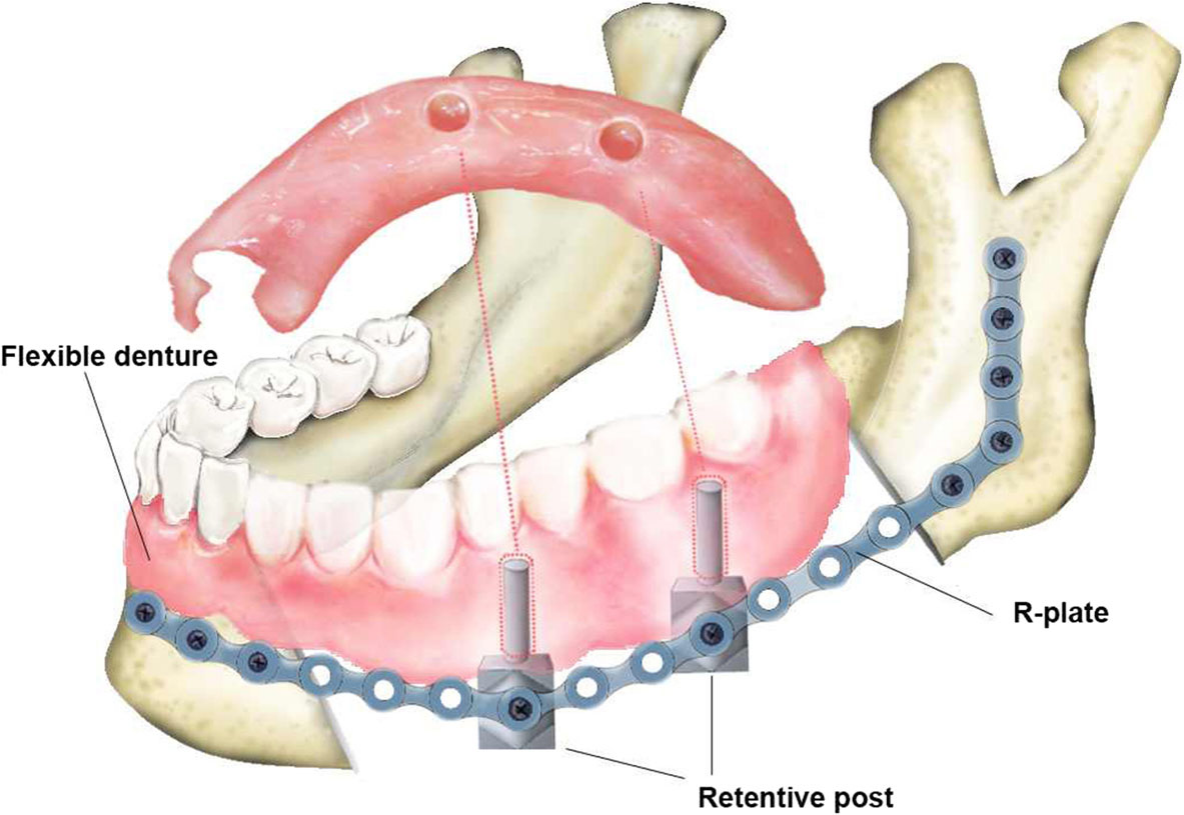
A representative schematic drawing of the mandibular reconstruction with retentive posts and flexible denture

Recently, we suggested a new technique for functional mandibular reconstruction using retentive posts of R-plates and flexible dentures. These newly designed retentive posts were fabricated using titanium metal like that in the R-plate. Virtual simulations using the finite element method (FEM) helped to design and optimize the retentive posts. Two or three retentive posts per quadrate jaw were fixated on the R-plate. Flexible dentures, made of thermoplastic resin, were used as removable prostheses to rehabilitate the occlusion successfully (Fig. 1). The patients were able to undergo functional rehabilitation without any additional bone grafts or major surgeries. The results from two patients, over the course of >3 years, are described in this article.

## Methods

### Fabrication of retentive posts for the reconstruction plate

**Fig. 2:**
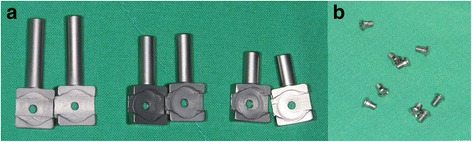
Retentive posts including the retention part and connection part, 2.0, 1.5, and 1.0 cm in length (**a**) and screws for connecting the posts (**b**)

A Leibinger® mandibular R-plate (2.8 mm height) and 2.7 mm locking screws were used for the primary mandibular reconstruction. Newly designed retentive posts of the R-plate were made with the same titanium materials suitable for adaptation to the existing R-plate with 2.0-mm locking screws (Fig. 2). The posts consisted of the connection and retention parts. The retention part is a square pole shape and has three lengths: 1 cm short, 1.5 cm medium, and 2 cm long (Fig. 2a). It is exposed through the mucosa and serves as a retention for the prosthesis. The connection part links to the R-plate with a screw (Fig. 2b), which is square in shape and connects to each hole in the R-plate.

### FEM analyses

**Fig. 3:**
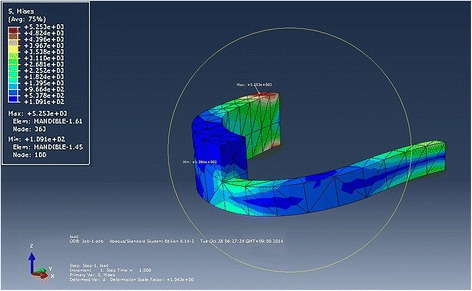
FEM analysis with the von Mises stress distribution in the mandible without retentive posts

**Fig. 4:**
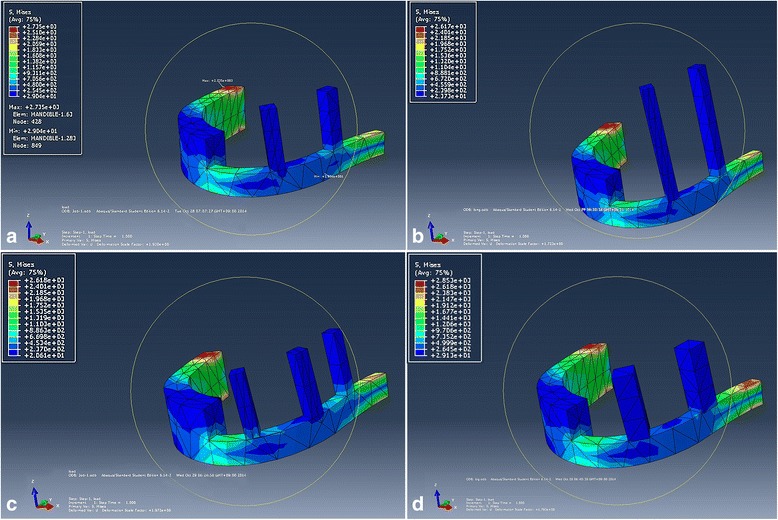
FEM analyses with von Mises stress distribution in the mandible inserted with various design retentive posts for R-plate, standard (**a**), long (**b**), three (**c**), and wide (**d**)

We were able to predict the stress and strain of the retentive posts by simplifying the geometric FEM model. The FEM model is a numerical method that creates models that approximate reality. A horseshoe-shaped simplified mandibular model was designed. The R-plate was inserted as a thinner rectangular cross section plate, and the retentive posts of the R-plate were designed as square pole shapes of various numbers, lengths, and widths (Figs. 3 and 4).

Analyses were performed using the FEM program Abaqus® (version 6.14, SIMULIA Co., RI, USA) and ImageQuant® (Version 5.2, Molecular Dynamics Co., USA). We set the model to only include muscles that can transmit traction, and joint areas that can transmit pressure. The mechanical properties of the involved materials were characterized as isotropic, homogeneous, and linearly elastic. Based on a previous study [6], the elastic modulus of cortical bone and the R-plate were set to 105,000 and 8700 N/mm^2^, respectively. Poisson’s ratio was set at 0.3 for both. The vertical chewing force was 135 N (kg m/s^2^). These properties were evenly applied to the upper face of the mandible and all posts (Table 1). Comparisons between different numbers, lengths, and widths were performed with ANOVA using SPSS for Windows® (Version 12.0, SPSS Inc., USA). The mean values are followed by 95% confidence intervals.

**Table 1:** The maximum stress according to von Mises stress in the various retentive post systems

Modification	Stress at Rt. TMJ (N/mm^2^)	Stress at end of Lt. R-plate (N/mm^2^)	Stress at connection of R-plate and mandible (N/mm^2^)	Stress at connection of R-plate and post (N/mm^2^)
No post	5253	3538	2681	–
Standard	2735	2284	1157	705.6
Long	2617	2185	1104	672.0
Three	2618	2185	1103	1103
Wide	2853	2618	1206	499.9

### Adaptations to the patients

**Fig. 5:**
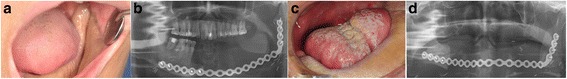
Clinical photos of intraoral (**a**) and panoramic view (**b**) of case 1 patient, and another intraoral (**c**) and panoramic view (**d**) of case 2 patient

**Fig. 6:**
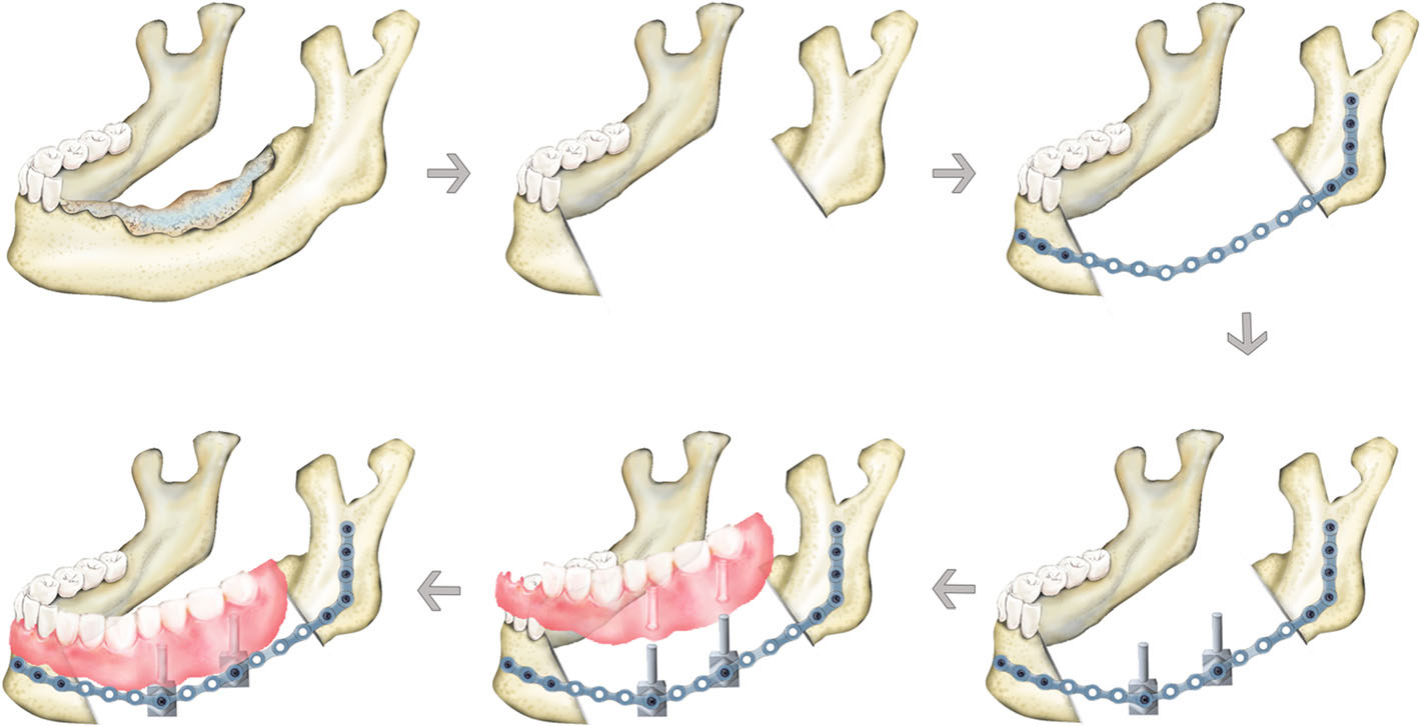
A whole sequential diagram, including partial mandibulectomy, reconstruction with R-plate, retentive posts adaption, and prosthesis fabrications

Among total eight patients treated with our retentive posts and prostheses, representative two patients with a diagnosis of oromandibular malignancy were summarized in Table 2. Unfortunately, three patients were expired due to their aggravated general conditions, and another two patients were not finished prosthetic procedures. So we used only two patients after their 3 years’ follow-up. A statement of ethics approval in the department of oral and maxillofacial surgery at Seoul National University Dental Hospital was approved by our institutional review board. First, both patients underwent massive tumor ablation and simultaneous reconstruction with LD myocutaneous free flaps and R-plates (Stryker Co., Kalamazoo, MI, USA) (Fig. 5). After waiting more than 2 or 3 years’ follow-up without any recurrences, both patients underwent a second surgery for the fixation of the retentive posts under local anesthesia. When the wound was completely healed, prosthodontic procedures were initiated. Figure 6 is a diagram of the adaptation procedures, including partial mandibulectomy, reconstruction with R-plate, retentive posts adaption, and prosthesis fabrications.

**Table 2:** Clinical profiles of two patients in this article

Case	Age	Sex	Histology	Location	Stage	Adjuvant therapy	Interval between primary surgery and post adaptation	Post design	Prosthesis
1	58	F	MM	Lt Mn	pT4aN1M0	PORT	2 years and 3 months	Two 1.5 cm long	Flexible denture
2	52	M	SCC	Lt Mn	pT4aN0M0	PORT	3 years	Three 1.0 cm long	Flexible denture

*MM* malignant melanoma, *SCC* squamous cell carcinoma, *Lt* left, *Rt* right, *Mn* mandible, *PORT* post-operative radiation therapy

**Fig. 7:**
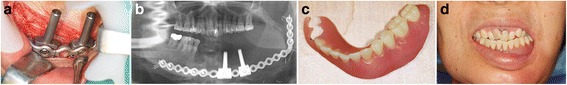
Clinical photos of case 1 patient showing elevation of skin pedicles and adaptation of retentive posts (**a**), post-insertional panoramic view of two retentive posts (**b**), flexible denture for prosthesis (**c**), and final appearance of patient (**d**)

**Fig. 8:**
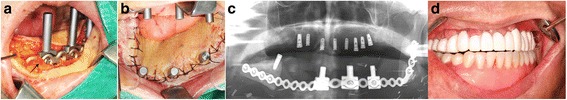
Clinical photos of case 2 patient showing elevation of skin pedicle and dissection without injury to the small musculocutaneous perforators (**a**, *black arrow*), intraoral suturing and exposure of three posts (**b**), post-insertional panoramic view of three retentive posts (**c**), and final appearance of wearing denture (**d**)

Both patients underwent similar surgeries under local anesthesia. An incision was made between the vestibule mucosa and the previously grafted LD skin. A skin pedicle was elevated and dissected toward the R-plate (Fig. 7a and 8a), while avoiding any injury to the small musculocutaneous perforators (Fig. 8a with black arrow). After exposing the R-plate, the position of the retentive posts was confirmed according to the ideal location of FEM. Two or three retentive posts were also connected with screws, according to the defect length and individual intermaxillary vertical dimension. After finishing the post adaptation under the 35 Ncm with a torque controller (3i Co., FL, USA), the elevated flap was carefully sutured with 4-0 Vicryl® (Polyglactin 910, Johnson & Johnson Co., USA). The post was left exposed in the oral cavity (Figs. 7b and 8b, c).

### Prosthodontic adaptation with flexible denture and resin teeth

Flexible dentures were made using Valplast® partial dentures (Valplast International Co., Westbury, NY, USA). The polyamide resin, developed from a type of nylon material, has a lower elastic modulus than do acrylic resins. Therefore, the polyamide resin is soft, easily deformable, and elastic [7]. Flexible dentures use undercuts to achieve retention and stability without a metal clasp (Fig. 7c). Therefore, flexible dentures are lightweight and easier to apply than are other dentures. In order to avoid any strong occlusion forces, the resin teeth were made not with gold materials but only with acrylic resins.

## Results

### Design and optimization of retentive posts

The von Mises stress distribution in the R-plate reconstructed mandible (with retentive posts of different numbers and lengths) was designed in the FEM analysis (Fig. 3). Four types of posts were simulated for optimized design and number, with comparisons between standard and long, two and three, and two wide. The long posts seemed to have similar amounts of stress and distribution compared to those of the standard length (Fig. 4a, b). Three posts had additional stress at the posterior post (Fig. 4c), and the last one had an increased stress point at the posterior R-plate boundary in the wide design (Fig. 4d, Table 1).

Stress was concentrated at a connection point between the mandible and R-plate. As two standard posts were adapted, a small amount of additional stress was loaded to the R-plate posteriorly (Fig. 3). As posts were adapted, stress was distributed at posts and additional concentration was loaded at a connection point of R-plate and posts (Fig. 4). Although our trial acquires statistical correlation values between these different numbers, lengths, and widths, one modeling of each of the four types cannot be compared with statistical significance. However, it has significantly more stress than that occurring without the post. The length of the post did not significantly affect the amount of stress. Based on these stress distributions, one can choose between two and three posts according to the patient’s arch length. Each post length can then be adapted after checking the occlusional clearance.

### Prosthodontic procedures

After the surgical wound healed completely, between 4 and 6 weeks, the prosthodontic procedures were initiated. A total of four steps were required: (1) preliminary, (2) final impression, (3) wax denture fitting, and (4) delivery of final prosthesis.

First, a preliminary impression was taken to make the individual tray. The model was made with yellow stone. The individual tray was fabricated using resin materials, which were also used for the final impression. The final impression was made using this individual tray and rubber impression materials. A bite registration was also performed. The final stone model was made and sent to a dental technician for denture fabrication. A wax denture fitting was used to confirm the bite status and appearance of the final prosthesis. The wax denture was again sent to the dental technician, and the final prosthesis was made based on this fitted wax denture. Finally, a flexible denture was fabricated for both patients (Figs. 7d and 8d). A resin tooth-fixed prosthesis of upper maxilla was fabricated for the second patient too. These prostheses were delivered to the patients and adjusted as appropriate and close cooperation with a dental technician is essential for the successful treatment.

### Case 1

The patient is a 58-year-old woman with an exophytic black pigmented lesion on the lower left gingiva. The lesion was 16 mm long with buccal cortex invasion. It was diagnosed as a malignant melanoma. Extensive mass excision was performed with partial mandibulectomy, functional neck dissection with level I to III, reconstruction with an R-plate and LD, and tracheostomy. Given the large size of the primary lesion and metastatic lymph nodes, post-operative radiotherapy with 7200 Gray and additional chemotherapy were performed (Fig. 1a, b and Table 2).

There was no recurrence after 2 year and 3 months. At this time, two posts were applied under local anesthesia (Fig. 7a). The final position and integrity of the posts were confirmed on a panoramic view (Fig. 7b). After several stepwise fabrication procedures, the final flexible denture (Fig. 7c) was delivered to the patient, and the patient has been wearing the denture for the last 3 years and experienced improved lip support by denture without other serious complications, as well as tolerable functions including chewing, swallowing, and pronunciation (Fig. 7d).

### Case 2

The patient was a 52-year-old man with a rapidly enlarging ulcerative mass on the left lower gingiva. The lesion invaded into several muscles and the marrow of the mandible. It was diagnosed as a squamous cell carcinoma. Partial mandibulectomy was performed under general anesthesia, with functional neck dissection of level I to III, reconstruction with an R-plate and double free flap (radial forearm free flap and LD), and tracheostomy. Although metastatic lymph nodes were not found, post-operative radiotherapy was planned for an inadequate surgical margin (Fig. 1c, d and Table 2).

During 3 years of follow-up, there was no recurrence or metastasis. Therefore, post adaptations were planned. Three posts of 1.0 cm in length were inserted (Fig. 8a, b). Additional dental implants were also installed into the maxilla for the upper denture (Fig. 8c). Both upper and lower prosthodontic procedures were planned simultaneously, concerning the patient’s edentulous arch, lack of vestibular mucosa, and favorable occlusal relations (Fig. 8d). The patient had a chewing occlusion and improved masticatory force without other serious complications during the last three and half years.

## Discussion

It is challenging to reconstruct a mandible that has already been reconstructed with an R-plate. Although this can be solved with bone grafting, patients may not be in the condition to tolerate another major surgery. There are several reasons that make bone grafting difficult, or even impossible. Neck dissection, followed by soft tissue free flap surgery, makes additional osseous free flap surgery very difficult. This results from poor recipient vessel status. Radiation therapy makes additional reconstruction even more difficult, because it induces soft tissue fibrosis and changes the vessel; and previous radiation therapy increases the risk for a complicated or failed flap procedure [8]. Therefore, patients with a risk factor for additional osseous flap surgery are suitable for indication of our suggested retentive posts. Furthermore, medical compromise that prevents the use of general anesthesia is another absolute indication of these retentive posts (Table 3). Our newly designed retentive posts with flexible dentures (Fig. 1) offer an alternative treatment for final functional rehabilitation in these patients.

**Table 3:** Inclusion criteria for retentive posts on reconstruction plates

Inclusion criteria
1. Difficulty of additional free flap surgery
1.1. Previous free flap surgery
1.2. Previous neck dissection
1.3. Radiation therapy
2. Medically compromised patients
3. Patients unwilling to undergo additional major surgery

Retentive posts can be designed with variable numbers, lengths, and widths. We attempted to optimize the design and post-insertional changes of the stress distribution of the R-plate with FEM analysis. After receiving major ablation surgery combined with long reconstruction plate with large-volume’s free flap, every patient did not want to receive more reconstruction surgery such as osteocutaneous free flap. In these cases, we have designed the retentive posts with different lengths and have applicated two or three posts to the reconstruction plate directly, based on preliminary FEM analysis with reconstructed mandibular model. From our tentative analysis, we could confirm more than two posts and short posts were available and a connection point between the remained mandible and reconstruction plate must be avoided due to its high stress concentration. Thus, after decision of the first location in the anterior region, second or even third post could be located according to occluding maxillary arch type.

Stress on a mandible without any posts is only loaded on the bone itself. Stress on the R-plate is smaller than that on a mandible with inserted posts (Fig. 3). Specifically, there is an increased stress at the connection between the post and plate, as well as the condylar portion of the R-plate (Fig. 4a). The length of the post does not affect the stress distribution (Fig. 4b). Therefore, the post length can be determined by the intermaxillary distance and interocclusal clearance. The number of posts is also an important consideration. When comparing plates with two and three posts, there is increased stress at the connection of the third post and plate (Fig. 4c and Table 1). Therefore, for the quadrant jaw, two posts are suitable, while a third post increases the stress. After determining the length and number, the width of the posts should be considered. We simulated two kinds of posts with different width design, and there was increased stress at the rearmost area around the condyle side (Fig. 4d). Wide posts can add unnecessary stress to the R-plate. And finally, we conclude that two regular-sized posts are appropriate for quadrant jaw reconstruction and the length of the posts should be determined based on the intermaxillary distance and prosthesis height.

Although mandibular reconstruction with an R-plate is popular, the technique still has several complications. Exposure or R-plate fracture is the most frequent complication, which predominantly occurs in patients with a history of radiation therapy [9]. Fortunately, these complications were not observed in our cases, which involved pre-radiation patients. Screws connected to the posts can be loosened through continuous occlusional loading. We have observed screw loosening in our previous case, so we tightened the screws during post modification surgery. This expected complication could be minimized by using a torque controller during the first tightening procedure.

In most cases of R-plate reconstruction of mandibular continuity, there were no attached gingiva. The mucosal vestibule was also lacking because a skin pedicle or movable gingiva covered the R-plate. These poor oral conditions make it difficult for a patient to appropriately clean around the retentive posts and prosthesis. This can eventually lead to gingival inflammation and pain. Most of our previous patients, including these two cases, had gingival inflammation around the retentive post at their first use. Difficulty of cleaning the posts and prosthesis can potentially require its removal and adjustment. Once a patient can perform self-irrigation around the posts, inflammation decreases significantly. Therefore, patients should be educated periodically regarding self-cleansing in order to prevent these complications.

Recently, computer-assisted design and manufacturing have become popular with three dimensional printing technology for mandibular reconstruction. Many surgeons and researchers have developed various patient-specific R-plates for mandibular reconstruction [10]. Designs can be optimized using FEM, which simulates the stress distribution on the plate [11]. This technology offers various opportunities and advantages over those of traditional R-plates and these could be applied to many cases of mandibular reconstruction. Our newly designed posts, which are adapted to the existing R-plate, represent the first step toward functional and esthetic mandibular reconstruction with R-plate. Although this article has the limited patients number, patient-specific R-plates with retentive posts will be applied in place of conventional methods from this preliminary study in the near future. Retentive posts can be imbedded into the patient-specific R-plate at the optimal position and design. This will not only decrease post-operative complications, but also offer improved esthetic appearances with function rehabilitation including chewing and swallowing.

## Conclusions

In post-mandibulectomy patients, retentive posts of the R-plate and upper flexible prostheses can allow functional dental rehabilitation without the need for bone grafts. These methods produced final dental occlusion in patients without additional major surgeries for bone grafts. Virtual preliminary simulation using FEM can help to optimize the design and number of retentive posts. Two or three regular-sized posts are suitable for the quadrant jaw. Based on these preliminary outcomes, improved patient-specific rehabilitation could be expected in the near future.
